# Efficacy of Fluidized Bed Bioartificial Liver in Treating Fulminant Hepatic Failure in Pigs: A Metabolomics Study

**DOI:** 10.1038/srep26070

**Published:** 2016-05-19

**Authors:** Pengcheng Zhou, Li Shao, Lifu Zhao, Guoliang Lv, Xiaoping Pan, Anye Zhang, Jianzhou Li, Ning Zhou, Deying Chen, Lanjuan Li

**Affiliations:** 1 State Key Laboratory for Diagnosis and Treatment of Infectious Diseases, Collaborative Innovation Center for Diagnosis and Treatment of Infectious Diseases, The First Affiliated Hospital, School of Medicine, Zhejiang University, Hangzhou, Zhejiang 310003, P.R. China; 2 Xiangya Hospital, Central South University, Changsha, Hunan 410008, P.R. China

## Abstract

Bioartificial livers may act as a promising therapy for fulminant hepatic failure (FHF) with better accessibility and less injury compared to orthotopic liver transplantation. This study aims to evaluate the efficacy and safety of a fluidized bed bioartificial liver (FBBAL) and to explore its therapeutic mechanisms based on metabolomics. FHF was induced by D-galactosamine. Eighteen hours later, pigs were treated with an FBBAL containing encapsulated primary porcine hepatocytes (B group), with a sham FBBAL (containing cell-free capsules, S group) or with only intensive care (C group) for 6 h. Serum samples were assayed using ultra-performance liquid chromatography-mass spectrometry. The difference in survival time (51.6 ± 7.9 h vs. 49.3 ± 6.6 h) and serum metabolome was negligible between the S and C groups, whereas FBBAL treatment significantly prolonged survival time (70.4 ± 11.5h, *P* < 0.01) and perturbed the serum metabolome, resulting in a marked decrease in phosphatidylcholines, lysophosphatidylcholines, sphingomyelinase, and fatty acids and an increase in conjugated bile acids. The FBBAL exhibits some liver functions and may exert its therapeutic effect by altering the serum metabolome of FHF pigs. Moreover, alginate–chitosan capsules have less influence on serum metabolites. Nevertheless, the alterations were not universally beneficial, revealing that much should be done to improve the FBBAL.

Hepatic failure is one of the most serious diseases, with a high risk of mortality encountered in clinical practice^1^. The only long-term effective treatment is orthotopic liver transplantation^2^,^3^, and this approach has been limited by the scarcity of donor livers. As an alternative, artificial livers have demonstrated good performance in combating metabolic disturbance and improving the clinical situation of hepatic failure patients^2^,^3^. However, these non-biological livers have achieved limited success, presumably because of their insufficient replacement of the synthetic and metabolic liver functions^4^,^5^,^6^. Therefore, it is urgent to develop new approaches to improve the management of hepatic failure.

Many researchers have attempted to develop extracorporeal bioartificial liver (BAL) systems that incorporate living and functional hepatocytes into bioreactors to provide functions similar to the *in vivo* liver. Recently, a new BAL-fluidized bed bioartificial liver (FBBAL) has been developed by our group^7^. The system incorporates encapsulated primary porcine hepatocytes into a choanoid fluidized bed bioreactor. It is believed that such a structure of the bioreactor will facilitate bidirectional mass transport during BAL treatment and provide a favorable environment for maintaining the function of hepatocytes^7^,^8^,^9^. In a preclinical test, the FBBAL was demonstrated to prolong the survival time and improve certain blood biochemical parameters, such as lactic acid, glucose and the Fisher index, in fulminant hepatic failure (FHF) pig models^7^. Therefore, the FBBAL might be a promising choice for the clinical treatment of end-stage liver diseases.

However, problems remain that preclude the application of this technology. Firstly, FBBAL relies on alginate–chitosan encapsulation, but its influence on serum during treatment has not been studied. Secondly, the efficiency and therapeutic mechanisms of FBBAL remain to be revealed. Considering that the liver is the main organ of metabolism and that hepatic failure always results in serious disturbance of endogenous metabolites^10^, it would be useful to study the performance and therapeutic mechanisms of an FBBAL with respect to metabolism. Nevertheless, most previous research has focused on a few metabolic parameters due to the limits of technology^11^ and has thus revealed limited information.

The emergence of metabolomics has made it possible to investigate the overall metabolome of a biological system simultaneously^12^ and therefore will provide promising strategies for the study of BALs in depth. For example, Hao *et al*. characterized the plasma metabolome of hepatitis B virus-induced acute-on-chronic liver failure after non-biological liver treatment using ultra-high-performance liquid chromatography-mass spectrometry (UPLC-MS) and developed a prognostic model that is more accurate than end-stage liver disease scores in predicting the potential need for liver transplantation for hepatic patients undergoing artificial liver support treatment^13^.

Here, our aim was to characterize the performance of FBBAL treatment in depth using UPLC-MS combined with multivariate statistical analyses to achieve the following three objectives: confirming the efficacy of FBBAL treatment based on survival analysis, revealing the impacts of alginate–chitosan encapsulation by comparing the survival time and serum metabolome between pigs in a sham FBBAL group (S group) and those in an FHF group (C group), and revealing the therapeutic mechanisms of the FBBAL by comparing the serum metabolome in the FBBAL group (B group) with the metabolome in the S group after treatment. The results will be of great help in accelerating the improvement of FBBALs.

## Results

### FBBAL treatment prolonged the survival time of FHF pigs

**Figure 1:**
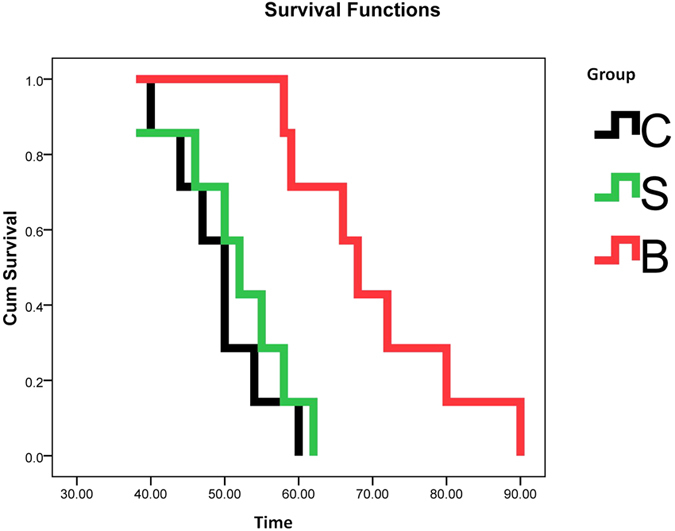
Survival curves. FBBAL treatment significantly prolonged the survival time. Survival time for group B, 70.4 ± 11.5 h; group S, 51.6 ± 7.9 h; and group C, 51.6 ± 7.9 h. B group vs. S group, *P* < 0.01. B group vs. C group, *P* < 0.01. S group vs. C group, *P* = 0.637.

As shown in Fig. 1, all pigs in groups C (49.3 ± 6.6 h) and S (51.6 ± 7.9 h) died nearly 50 h after FHF induction, whereas the survival time of group B was approximately 70 h (70.4 ± 11.5 h, *P* < 0.01). Moreover, the survival time between groups C and S was not significantly different (*P* = 0.637). Furthermore, there were no statistically significant differences between the groups in terms of biochemical indicators (Supplementary Table S1). The results indicated that FBBAL treatment could prolong the survival time of FHF pigs, confirming the favorable therapeutic effect of FBBAL in combating FHF.

### Pigs in different groups had comparable metabolomes before treatment

**Figure 2:**
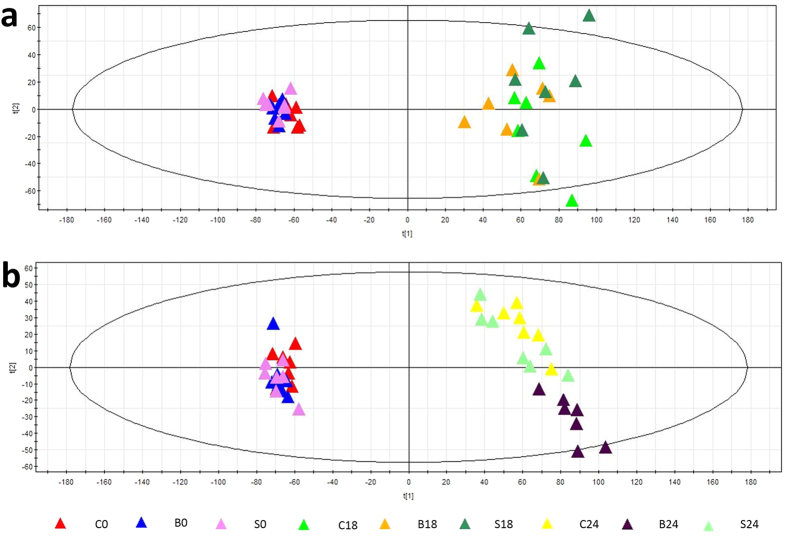
PCA score plots. (**a**) The PCA score plot of samples at 0 h and 18 h. The 0 h samples of all three groups (C0, S0, B0) are clustered on the left. The 18 h samples of all three groups (C18, S18, B18) are located on the right, and the 18 h samples of the three groups are intermixed. (**b**) The PCA score plot of samples at 0 h and 24 h. After treatment (24 h), groups C and S were still intermixed, but group B clustered distinctly from the other two groups in the PCA score plot.

As shown in Fig. 2a, the 0 h samples of all three groups (C0, S0, B0) are clustered in the left in the PCA score plot; the 18 h samples of all three groups (C18, S18, B18) are located in the right of the plot, and the 18 h samples of the three groups are intermixed with each other. Furthermore, the correlation coefficients of the 0 h and 18 h samples in all groups were 0.91 ± 0.06 and 0.91 ± 0.07, respectively, as shown in the heat map (Supplementary Fig. S1a,b). The results demonstrated that there were no apparent metabolome differences among pigs in different groups in this study.

### Serum metabolome was altered significantly after the introduction of FHF

**Figure 3:**
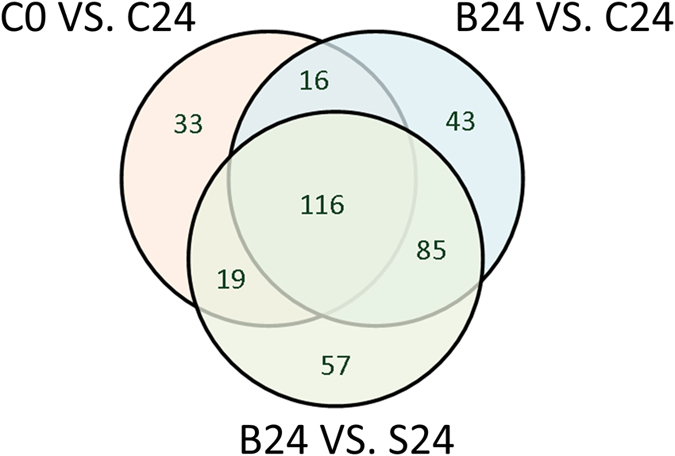
The proportion of shared regulated metabolites resulting from the induction of FHF, FBBAL treatment and the metabolic effect of hepatocytes. Significantly changed metabolites resulting from the induction of FHF: metabolites with VIP > 1 in the PLS-DA model (group C at 0 h vs. group C at 24 h), marked as C0 vs. C24. Significantly changed metabolites due to FBBAL treatment: metabolites with VIP > 1 in the PLS-DA model (group B at 24 h vs. group C at 24 h), marked as B24 vs. C24. Significantly changed metabolites due to the metabolic effect of hepatocytes: metabolites with VIP > 1 in the PLS-DA model (group B at 24 h vs. group S at 24 h), marked as B24 vs. S24.

As shown in Fig. 2a,b, the PCA score plot exhibited distinct clustering before the administration of D-gal (group C at 0 h) and after its administration (group C at 18 h and 24 h). As shown in Fig. 3, for controls (group C at 0 h), the development of FHF (group C at 24 h) significantly altered 184 metabolites (variable importance in the projection (VIP) > 1, marked as C0 vs. C24). The results indicated that the serum metabolome was significantly perturbed after FHF induction.

### Alginate–chitosan capsules had little influence on serum metabolites

After treatment (24 h, n = 7), group C at 24 h and group S at 24 h were still intermixed (Fig. 2b). No significant PLS-DA model or metabolites could be discerned based on the above two groups. As shown in the heat map (Supplementary Fig. S1c), the correlation coefficients (similarity) between samples in group C at 24 h and group S at 24 h were close to one (0.94 ± 0.05). The results confirmed that cell-free alginate–chitosan capsules in the FBBAL had little influence on the serum metabolites of FHF pigs.

### Therapeutic effect of FBBAL on the metabolome

After treatment for 6 h, the FBBAL treatment group (group B at 24 h) aggregated further from the FHF (group C at 24 h) and the sham treatment (group S at 24 h) groups and formed a new, separate cluster (Fig. 2b), indicating that the serum metabolome of FHF pigs might have been altered by FBBAL treatment.

As presented in Fig. 3, the comparison between group C at 24 h and group B at 24 h indicated that FBBAL treatment dramatically regulated 260 metabolites (VIP > 1 in the PLS-DA model, denoted as B24 vs. C24), and these regulated metabolites might result from the overall effect of the hepatocytes and the alginate–chitosan capsule. Therefore, the metabolites altered in FBBAL treatment (with hepatocytes), excluding those regulated by alginate–chitosan capsules (without hepatocytes), indicate the potential metabolic effect of hepatocytes. Upon closer inspection, we found that 277 metabolites were significantly altered in group B at 24 h compared to group S at 24 h (VIP > 1 in the PLS-DA model, denoted as B24 vs. S24). The efficacy of each PLS-DA model was satisfactory and confirmed the reliability of the above results (Supplementary Table S2).

As shown in Fig. 3, the three types of treatment (the introduction of FHF (C0 vs. C24), FBBAL treatment (B24 vs. C24) and the metabolic effect of hepatocytes in FBBAL (B24 vs. S24) shared 116 metabolites in this study. The introduction of FHF and the treatment of FBBAL shared 132 metabolites, accounting for 71.74% (132/184) of metabolites resulting from the development of FHF. Furthermore, 135 out of the 277 metabolites regulated by the metabolic effect of hepatocytes in FBBAL were identified as FHF related, accounting for most of the 184 metabolites (135/184, 73.37%) perturbed by FHF. Finally, the treatment of FBBAL and the metabolic effect of hepatocytes in FBBAL shared 201 metabolites, indicating that most (77.31%, 201/260) of the 260 metabolites regulated by FBBAL treatment resulted from the metabolic effect of hepatocytes. In summary, FBBAL treatment altered the serum metabolome of FHF pigs through the metabolic effect of hepatocytes.

### Identification and dynamic patterns of significantly regulated metabolites

**Figure 4:**
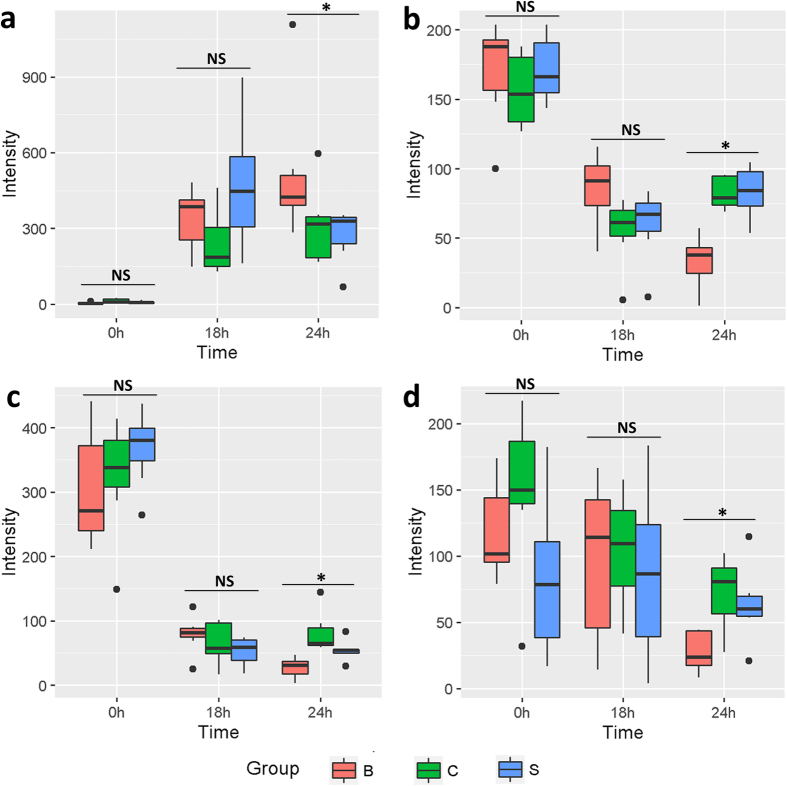
The dynamic changes in identified metabolites in each group at each time point. NS, No statistical difference; *P < 0.05. (**a**) GCDCA; (**b**) PC 16:0/18:2; (**c**) LPC 18:0; (**d**) linoleic acid.

After closer inspection, five types of metabolites were identified as FBBAL-regulated metabolites perturbed in FHF, including conjugated bile acids (CBAs), phosphatidylcholines (PCs), lysophosphatidylcholines (LPCs), fatty acids (FAs) and sphingomyelin (SM) (Table 1). CBAs were increased after the introduction of FHF, and then they were further elevated or delayed in their decrease after FBBAL treatment (Fig. 4a). Other metabolites, including PCs (Fig. 4b), LPCs (Fig. 4c), FAs (Fig. 4d) and SM, were decreased because of the introduction of FHF and further declined after FBBAL treatment. We also found that FBBAL treatment delayed the increase or decrease of certain metabolites. However, these metabolites could not be identified because of their low intensity in the UPLC-MS assay.

## Discussion

In this study, the therapeutic mechanism of an FBBAL was evaluated by comparing the metabolome of a pig model of hepatic failure in non-treated, FBBAL-treated and sham FBBAL-treated groups. Our results confirmed the efficacy of the FBBAL on FHF with respect to metabolomics and provide useful information for the further optimization of FBBAL systems.

Two important issues were considered in this study before any statistical analyses were carried out. Firstly, the stability and repeatability of resulting metabolomics data were revealed from the compact clustering of QC samples in the PCA score plot and the low CV of each identified metabolite in QCs^14^ (presented in the Materials and Methods section). Secondly, because three different groups of experimental pigs were utilized in this study, the within-group and between-group similarities, which were important to ensure the comparability of different groups, were investigated. According to the PCA and correlation analysis, there was no obvious difference in the serum metabolome among groups at 0 h (before the administration of D-gal) and 18 h (at the beginning of treatment). The above results certified the high quality of UPLC-MS data and good homogeneity of the pigs in different groups and thus support the reliability of the conclusions in this study.

Before any treatment can be applied in clinical settings, safety must be ensured. We evaluated the adverse effects of alginate–chitosan capsules in this study by comparing the untreated group and the group treated with sham FBBAL containing only empty capsules and found the following: the survival time of the pigs in the two groups was similar, and sham FBBAL treatment (S24) resulted in a similar metabolome to that of FHF (C24) receiving no treatment according to the information in the PCA score plot (Fig. 2a) and heat maps (Supplementary Fig. S1c). These findings indicated that cell-free alginate–chitosan capsules in the FBBAL had little influence on the serum metabolites of FHF pigs. In other words, the serum metabolomic changes after FBBAL treatment resulted from the metabolic effect of hepatocytes. Therefore, the alginate–chitosan capsules were proven safe with respect to their short-term effect on serum metabolites.

In addition to safety, efficacy is a primary concern that determines whether a treatment can be utilized in clinical settings. In this study, FBBAL treatment not only prolonged the survival time of pigs but also regulated most of the metabolic perturbations induced by FHF. In other words, FBBAL treatment exerts therapeutic efficacy by regulating the metabolites perturbed by FHF, especially those involved in bile acids, PCs, LPCs, and FAs metabolism. We also found that FBBAL treatment delayed the increase or decrease in certain metabolites in this metabolomic study, although the metabolites could not be identified because of their low intensity in the UPLC-MS assay. However, it should be pointed out that the levels of many regulated metabolites seemed to deviate from normal after FBBAL treatment.

With respect to the above metabolites that seemed to be abnormally regulated by FBBAL treatment, some of the alterations are reasonable or beneficial to a certain extent, as discussed below. Bile acid conjugation takes place in hepatocytes; thus, the increase in CBAs indicated that hepatocytes do play a role in the FBBAL; namely, hepatocytes can take in non-CBAs and transform them into CBAs^15^. However, FBBAL treatment occurs in the absence of a functional biliary excretion system^16^, which is the major method of clearance of bile acids. As a result, instead of clearance, serum CBAs were elevated after the FBBAL treatment. The decreasing trend in indirect bilirubins and constancy of total bilirubins after FBBAL treatment also support the above implications of bio-transformation^7^. PCs, necessary metabolites for the function of the hepatocellular membrane^17^, play a very important role in preventing the liver from being damaged^18^,^19^. Moreover, PCs can be broken down into choline and then facilitate the biosynthesis of GSH, an antioxidant in the body^20^. The most likely reason for the decrease might be that PCs are used to repair the injured hepatocytes and reduce the oxidative stress in FHF pigs during the FBBAL treatment. Considering the importance of PCs, the results strongly indicate that it is imperative to supply additional PCs to FHF patients when FBBAL treatment is performed. LPCs, as pro-inflammatory factors, induce pro-inflammatory cytokines and recruit immunocytes and thereby promote inflammation^21^,^22^. The decrease in LPCs after FBBAL treatment might down-regulate inflammation, thus prolonging the survival time of FHF pigs. Two reasons may account for their further decrease after FBBAL treatment. Firstly, as regulated by anti-inflammatory responses^13^,^23^, LPCs are cleared by enzymes (lysophospholipase and LPC-acyltransferase) that are located in hepatocytes in the FBBAL^24^. Secondly, the decrease in PCs, as the precursor of LPCs, might reduce the biosynthesis of LPCs. SM (d18:1/16:1) showed a similar trend as LPCs, which may be explained by the significant increase in sphingomyelinase (an enzyme located in hepatocytes) activity in acute toxic liver injury^25^. FAs are the major energy sources in stress^25^,^26^. The decline in FAs might result from the uptake of encapsulated primary porcine hepatocytes. Furthermore, FAs can be used to beta oxidize and provide energy to hepatocytes in the FBBAL to facilitate their function and transformed into ketone bodies or glucose in hepatocytes in the FBBAL and then transported to other organs of FHF pigs, such as the brain, as energy substances^27^. The relatively higher level of blood glucose after FBBAL treatment also supported this inference^7^.

Of course, these “deteriorative” changes in metabolites after treatment also indicate that the FBBAL is not perfect. Firstly, because the FBBAL does not have a toxic substance excretion system like the biliary tract system in the normal liver, which is a common problem for most bioartificial livers at present, it cannot imitate the function of the normal liver precisely. Furthermore, hepatocytes in the FBBAL might consume beneficial metabolites, such as PCs and fatty acids, to become active. Secondly, although it has been confirmed that FBBAL can biotransform and metabolize carbohydrates and fats, the clearance of toxic substances and biosynthesis should be validated or improved in future steps. Therefore, it is critical to improve the structure and function of the FBBAL in order to enable it to imitate the function of a normal liver as precisely as possible in the long run. At present, the combination of an FBBAL with non-bioartificial liver treatments, such as plasma exchange, plasma perfusion and albumin dialysis, might solve some of the problems and enhance its therapeutic effect. For example, plasma exchange prior to FBBAL treatment can clear toxic metabolites (CBAs, etc.) and increase beneficial metabolites (PCs, etc.), which would facilitate the functioning of hepatocytes in the FBBAL, thus enhancing the overall therapeutic effect.

In conclusion, it was confirmed in this study that alginate–chitosan capsules are safe with respect to their short-term effect on serum metabolites and that the FBBAL possesses some liver functions, such as biotransformation and metabolism, and exerts therapeutic effects by altering the serum metabolites perturbed by FHF. With respect to the finding that the alterations of metabolites are not universally beneficial, the FBBAL does not have a toxic substance excretion system, and hepatocytes in the FBBAL might consume beneficial metabolites. In summary, despite the efficacy of the FBBAL in treating FHF pigs, its structure and function still must be further upgraded before such technology can become the fundamental therapeutic approach for hepatic failure in the long run.

## Materials and Methods

### Chemicals

Acetonitrile, formic acid and leucine-enkephalin (HPLC grade) were purchased from Sigma (St. Louis, MO, USA). Distilled water was purified using a Milli-Q system (Millipore Bedford, MA, USA). Standard bile acids, fatty acids, phosphatidylcholines, lysophosphatidylcholines and sphingomyelins were purchased from Sigma-Aldrich (St. Louis, MO, USA).

### Animals and induction of FHF

The study was designed and carried out in accordance with the Chinese national research council guidelines and was approved by the Animal Ethical Committee of Zhejiang University. Male Chinese experimental miniature pigs (10–15 kg) were purchased from China Agriculture University (Beijing, China) and were housed in singular standard cages in an air-conditioned room (21–25 °C) with a 12-h light-dark cycle for 7 days before catheterization for adaptation. Standard laboratory chow and water were given ad libitum. Catheterization was performed by placing a catheter into the right jugular vein and artery of each pig with sterile techniques. FHF was induced in pigs via the intravenous administration of D-galactosamine (D-gal, 1.5 g/kg) (Hanhong Chemical Co., LTD, Shanghai) 24 h after the operation^7^,^28^.

### Hepatocyte encapsulation and FBBAL systems

**Figure 5:**
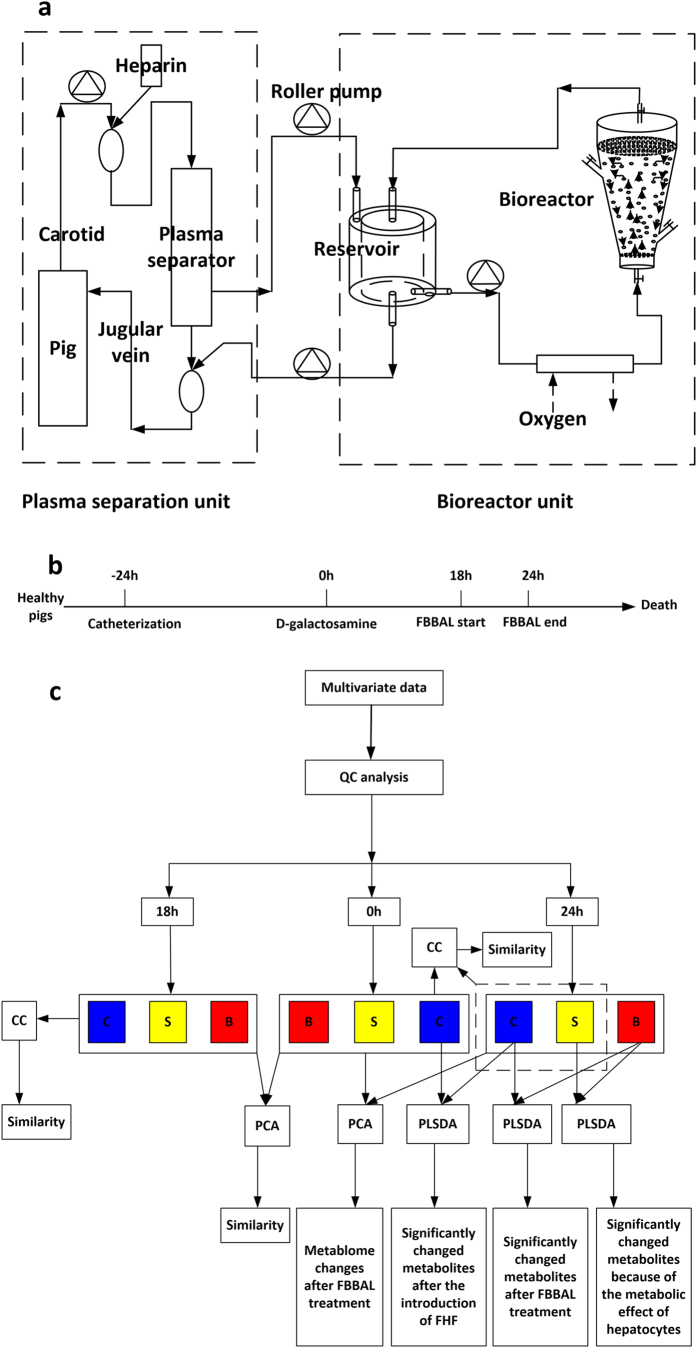
Schematic diagram of FBBAL treatment and data analysis. (**a**) Schematic diagram of FBBAL treatment. (**b**) Flow chart of the experiment. (**c**) Road map of multivariate data analysis.

The encapsulation of hepatocytes and constitution of FBBAL systems were detailed in our previous report^7^. Briefly, primary porcine hepatocytes were isolated using the four-step collagenase perfusion method^28^. The viabilities of the hepatocytes were up to 90%, as determined by trypan blue exclusion. The encapsulated primary porcine hepatocytes were obtained using the single-stage procedure alginate–chitosan encapsulation method^29^. The bioreactor (Chinese Patent No. ZL 200710070279.0) had a choanoid structure with a total volume of approximately 500 ml^29^. The BAL system consisted of a plasma separation unit and a bioreactor unit (Fig. 5a). Both units were driven by a series of roller pumps. All the equipment for the bioreactor unit were placed in an incubator maintaining an internal temperature of 37 °C when treatment was performed.

### Groups and FBBAL Treatment

In this study, 21 pigs were separated into the following three groups: (1) a FBBAL group (B group) in which pigs (n = 7) were treated with an FBBAL system containing encapsulated freshly isolated porcine hepatocytes (about 5 × 10^9^), (2) a sham FBBAL group (S group) in which pigs (n = 7) were treated with an FBBAL system with cell-free encapsulation and (3) an FHF group (C group) in which pigs (n = 7) were only given intensive care. Treatment was initiated 18 h after the administration of D-gal and lasted for 6 h^7^. The survival time of these pigs was determined from the time of administration of D-gal until the death of the pigs (Fig. 5b).

During FBBAL treatment, pigs were administered Diprivan and heparin (40U/kg/h) intravenously to maintain general anesthesia and to prevent blood clotting. Arterial blood was drawn from the pig at 18–25 ml/min and pumped into the plasma separator (OP-02W, Asahi-Kasei, Japan). The flow rate of the separating pump was controlled at 8–10 ml/min. The plasma reservoir (Chinese Patent NO: ZL 200920201694.X) served as an amortization device for the two units, enabling plasma to perfuse the bioreactor circularly at 25–35 ml/min. The purified plasma was returned to the pig body at the same rate as plasma separation.

### Sample collection and biochemical parameter assays

Blood samples were collected before the administration of D-gal (0 h), at the beginning of treatment (18 h) and at the end of treatment (24 h). At each time point, two portions of blood samples were collected and deposited in BD Vacutainer Plus Coagulation tubes and BD Vacutainer SST tubes (Becton, Dickinson and Company, Franklin Lakes, USA). After centrifugation at 3000 × *g* for 10 min at 4 °C, some of the serum (500 μl) and samples that were collected in BD Vacutainer Plus Coagulation tubes were sent to the clinical laboratory for biochemical parameters and blood coagulation (including aspartate aminotransferase, total bilirubin, total bile acid, creatinine and prothrombin time) assays. The remaining serum was aliquoted into fresh Eppendorf tubes and stored at −80 °C until the metabolomic assay.

### Sample preparation and UPLC-MS assay

All samples were thawed at 4 °C. Quality control (QC) samples were obtained by pooling aliquots (10 μl) of each serum sample^14^. Serum samples including QC samples were mixed with acetonitrile at a 1:3 ratio (v/v). Then, the mixtures were vortex mixed for 1 min and centrifuged at 14,000 × *g* for 10 min at 4 °C. The resulting clear supernatant was placed into UPLC vials and then stored at 4 °C before detection.

Chromatographic separations were performed on a Waters ACQUITY Ultra Performance LC system using an ACQUITY UPLC BEH C18 analytical column (i.d.2.1 mm × 100 mm, particle size 1.7 mm, pore size 130 Å). Water/formic acid (99.9:0.1, v/v) was used as mobile phase A and methanol/formic acid (99.9: 0.1, v/v) as mobile phase B with a flow rate of 300 μl/min. A linear gradient LC system (Waters, Milford MA) was optimized as follows: the composition of mobile phase B was changed from 3% to 80% at 7.5 min, then from 80% to 98% at 8 min, and was kept there for 5 min, and then it reached 100% at 0.5 min and was held for 3 min. The temperature of the sample manager was set to 4 °C and the injection volume was 2 μl for each analysis. The QC samples were injected at regular intervals (every 6 samples) throughout the analytical run.

Mass spectrometry was performed on a Waters Q-TOF Premier mass spectrometer operating in negative ion electrospray mode. The instrumental parameters were set as follows: mass range scanned from 50 to 1000, the MS acquisition rate was set to 0.3 s with a 0.02 s interscan delay, and high-purity nitrogen was used as the nebulizer and drying gas. The nitrogen drying gas was at a constant flow rate of 450 L/h, and the source temperature was 120 °C. The capillary voltage was set to 2.5 kV; the sampling cone voltage was set to 40.0 V. Argon was used as the collision gas, and the collision energy was set to 5.0 eV. MS/MS analysis was performed on the mass spectrometer set at different collision energies of 5–60 eV according to the stability of each metabolite. The time-of-flight analyzer was used in V mode and was tuned for maximum resolution (>10,000 resolving power at *m*/*z* 554.2615). The instrument was previously calibrated with sodium formate; the lock mass spray for precise mass determination was set by leucine enkephalin at *m*/*z* 554.2615 with a concentration of 0.5 ng/μl.

### Data preprocessing and statistical analysis

We used MarkerLynx Applications Manager Version 4.1 (Waters, Milford, MA, USA) to detect, integrate and normalize the intensities of the peaks to the sum of peaks within the raw UPLC-MS data of the sample. A multivariate dataset based on the retention time, *m*/*z* and signal intensity of the peaks was acquired, and multivariate statistical analysis was carried out using statistical software. Principal components analysis (PCA) and partial least squares discriminate analysis (PLS-DA) were carried out using SIMCA-P+ 12.0 software (Umetrics AB, Sweden). Correlation coefficients were obtained with Matlab Version 7.8 (R2009a) software, and heat maps were created using Cluster 3.0 software (University of Tokyo, Human Genome Center) and Java TreeView 1.1.6r2 software (http://jtreeview.sourceforge.net/). Detailed multivariate statistical analysis was carried out as described in the data analysis road map (Fig. 5c). One-way ANOVA, Kruskal-Wallis test and Kaplan-Meier analysis were conducted using SPSS v 16.0 software (SPSS Inc. Chicago, IL, USA). Differences were considered statistically significant at *P* < 0.05.

### Marker identification

Identification of compounds was achieved by comparing the mass spectra and retention index of potential biomarkers with authentic reference standards (Supplementary Fig S2). The HMDB (www.hmdb.ca) and METLIN (www.metlin.scripps.edu) databases were used to help with the identification.

### Quality control of UPLC-MS assay

The PCA score plot of all samples including QC samples illustrated that QC samples clustered well (Supplementary Fig S3). Close inspection revealed that the coefficient of variation (CV) of the relative intensity of each identified metabolite in QC samples ranged from 1.65% to 7.54%, with a median CV of 3.88% (Table 1). These results confirmed that the UPLC-MS assay in this study had excellent stability and repeatability^14^.

## Additional Information

**How to cite this article**: Zhou, P. *et al*. Efficacy of Fluidized Bed Bioartificial Liver in Treating Fulminant Hepatic Failure in Pigs: A Metabolomics Study. *Sci. Rep*. **6**, 26070; doi: 10.1038/srep26070 (2016).

## Supplementary Material

Supplementary Information

## Figures and Tables

**Table 1:** Significantly altered metabolites.

Retention Time *m*/*z*	Metabolites	Molecular ion	PLS-DA VIP Value			CV (%)
			C0-C24	B24-C24	B24-S24	
8.26_448.3084	GUDCA	M-H	18.0269	2.18458	4.5434	2.86
9.05_448.3082	GCDCA	M-H	6.49598	6.01778	4.95115	5.93
8.12_464.3035	GCA	M-H	9.50586	1.88202	5.55929	7.54
8.29_514.2848	TCA	M-H	3.82476	4.42131	5.04845	7.32
17.89_804.5808	PC 16:0/18:1	M+HCOOH-H	1.96226	3.68712	3.25785	3.8
17.39_742.5430	PC 16:0/18:2	M-CH3	2.21085	3.20904	3.02928	6.42
17.38_802.5633	PC 16:0/18:2	M+HCOOH-H	3.38055	4.58781	4.26791	4.47
17.19_850.5662	PC 16:0/22:6	M+HCOOH-H	2.08144	2.79293	2.1084	4.95
18.15_830.5971	PC 18:0/18:2	M+HCOOH-H	2.96766	4.13863	3.59226	3.52
18.82_832.6142	PC 18:0/18:1	M+HCOOH-H	1.7119	2.77195	2.2285	3.93
18.03_854.5970	PC 18:0/20:4	M+HCOOH-H	2.7317	3.34439	2.51558	3.54
17.29_826.5653	PC 18:2/18:2	M+HCOOH-H	2.27703	3.17148	2.43345	3.82
11.36_480.3082	LPC 16:0	M-CH3	7.55664	6.17569	4.43431	2.85
11.36_540.3318	LPC 16:0	M+HCOOH-H	3.05203	2.26561	1.47136	4.73
12.66_508.3387	LPC 18:0	M-CH3	6.14331	4.5241	2.92868	2.19
12.66_568.3637	LPC 18:0	M+HCOOH-H	2.7488	1.83116	1.15724	6.62
11.66_506.3245	LPC 18:1	M-CH3	4.88666	1.62669	3.11971	4.12
10.88_504.3086	LPC 18:2	M-CH3	5.54765	3.08174	3.81833	2.33
10.87_564.3338	LPC 18:2	M+HCOOH-H	2.20518	1.3016	1.57908	5.67
12.99_255.2316	Palmitic acid	M-H	2.37701	2.33908	2.05141	4.05
12.39_279.2305	Linoleic acid	M-H	3.01179	4.12476	2.99722	3.41
13.28_281.2460	Oleic acid	M-H	3.19431	3.97561	3.17768	3.42
16.43_685.5334	SM 18:1/16:1	M-CH3	1.14799	1.41793	1.27395	1.41
16.43_745.5554	SM 18:1/16:1	M+HCOOH-H	1.18176	1.27706	1.184	1.66

Abbreviations: GCDCA, chenodeoxycholic acid glycine conjugate; GUDCA, glycoursodeoxycholic acid; PC, phosphatidylcholine; LPC, lysophosphatidylcholine; SM, sphingomyelin.
