# Supplementary material for: Efficacy of Fluidized Bed Bioartificial Liver in Treating Fulminant Hepatic Failure in Pigs: A Metabolomics Study
Source: Sci Rep. 2016 May 19;6:26070. doi: 10.1038/srep26070 (PMC4872127; doi:10.1038/srep26070)
Supplement: Supplementary Information [file srep26070-s1.doc]

***Title：***Efficacy of Fluidized Bed Bioartificial Liver in Treating Fulminant Hepatic Failure in Pigs: A Metabolomics Study

***Authors：***Pengcheng Zhou, Li Shao, Lifu Zhao, Guoliang Lv, Xiaoping Pan, Anye Zhang, Jianzhou Li, Ning Zhou, Deying Chen, Lanjuan Li*

Supplementary Fig S1. Heat maps

a. The correlation coefficients heat map of the three groups at 0 h. As shown in the heat map, most of the area of the heat map is bright red, which indicates that nearly all the correlation coefficients were close to one (0.91±0.06).

b. The correlation coefficients heat map of the three groups at 18 h. As shown in the heat map, most of the area of the heat map is bright red, which indicates that nearly all the correlation coefficients were close to one (0.91±0.07).

c. The correlation coefficients heat map of group S and group C at 24 h. As shown in the heat map, most of the area of the heat map is bright red, which denotes that nearly all the correlation coefficients were close to one (0.94±0.05).


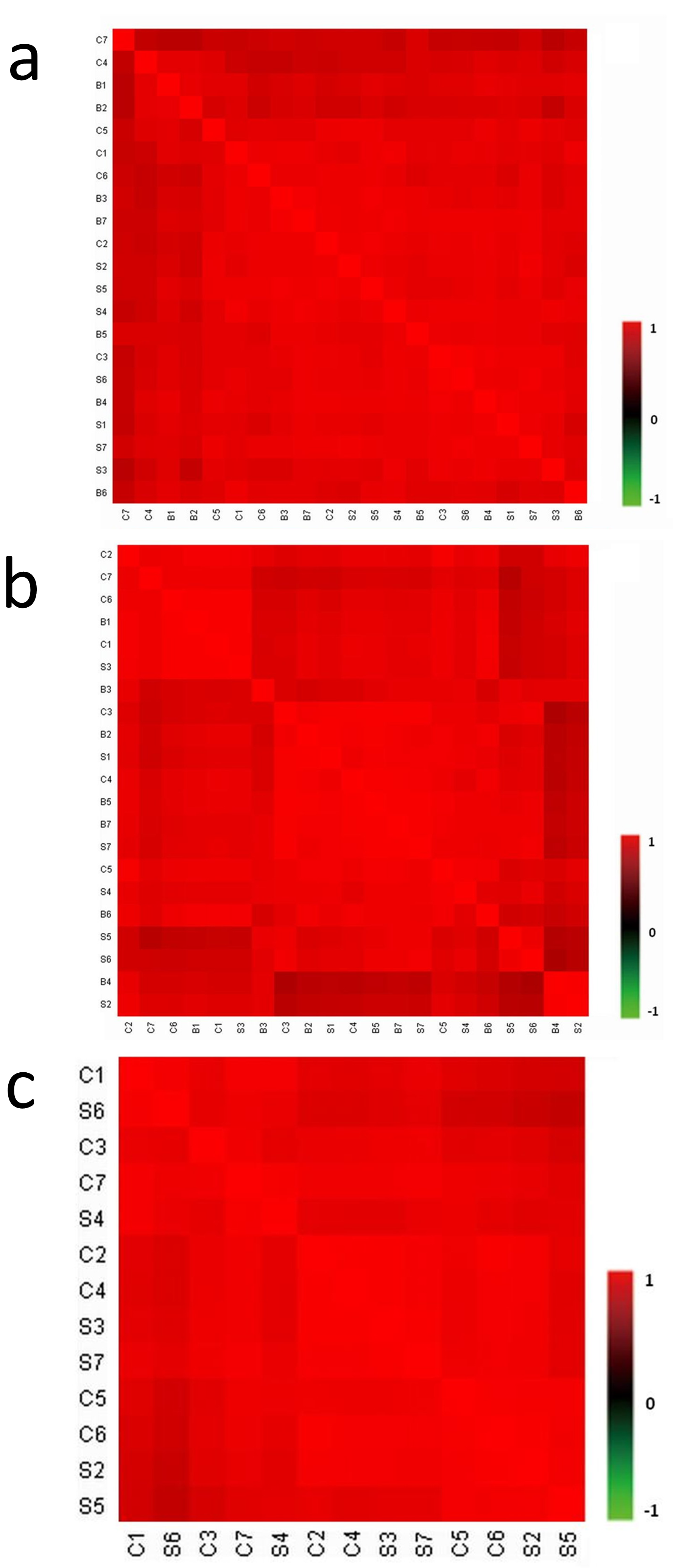


Supplementary Fig S2. MS/MS identiﬁcation of GCDCA.


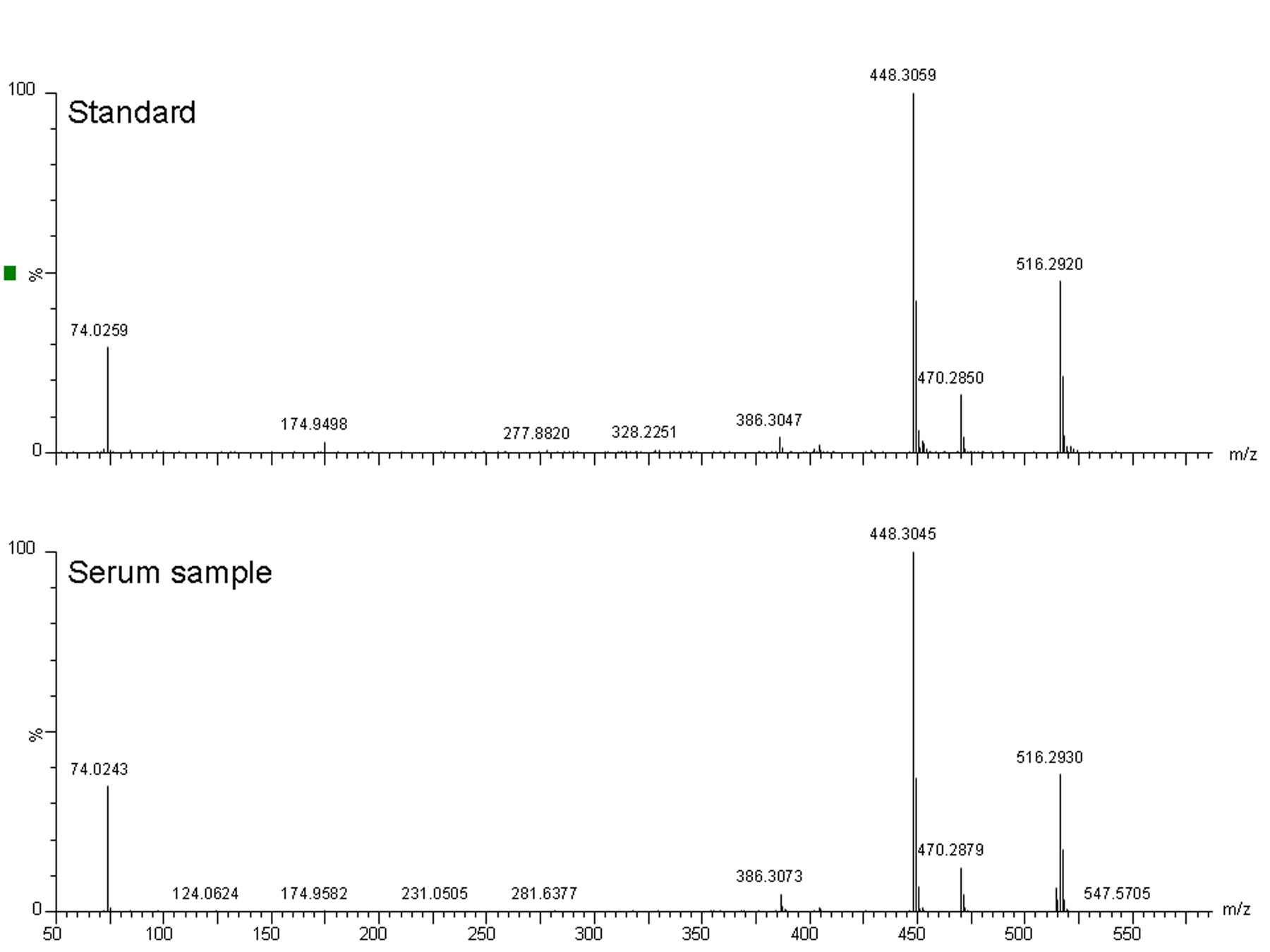


Supplementary Fig S3. The PCA score plot of all samples including QC samples. QC samples are clustered well and located in middle of the plot.


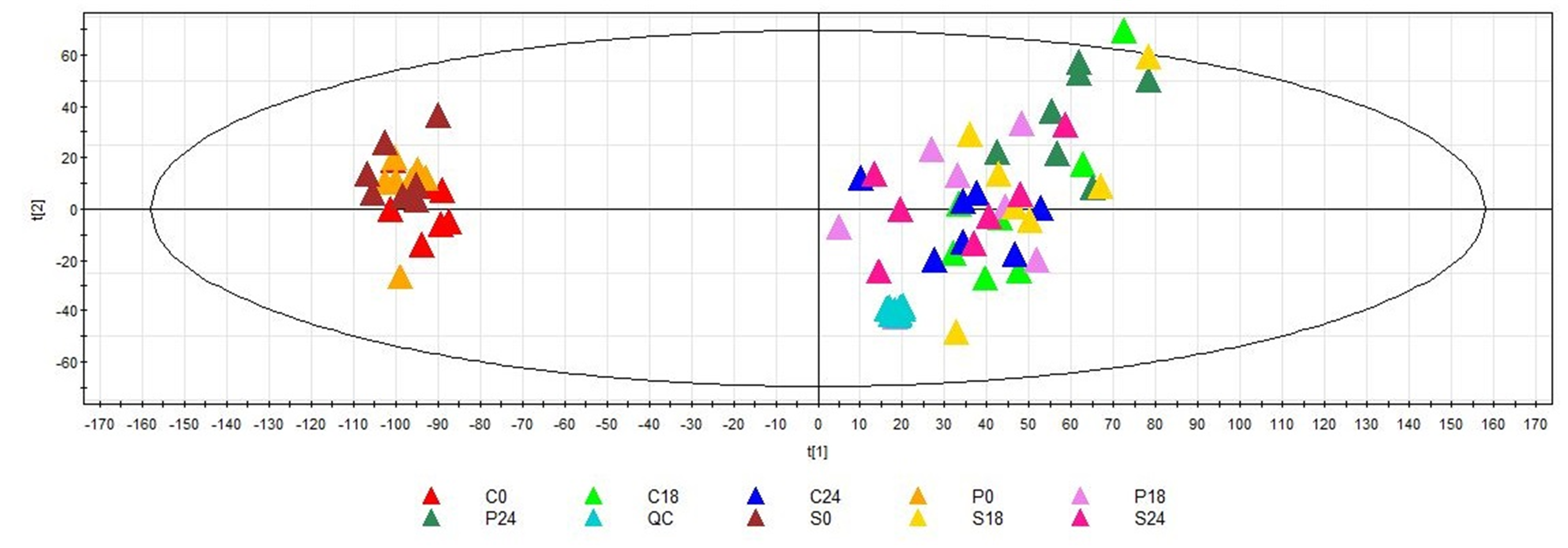


**Supplementary Table.S1**

**Blood biochemical parameters and blood coagulation**

|  |  | ALT(U/L) | TB(μmol/L) | TBA(μmol/L) | CR(μmol/L) | PT(s) |
| --- | --- | --- | --- | --- | --- | --- |
| 0h | C | 40.57±17.24 | 2.23±0.44 | 11.00±6.68 | 37.43±7.98 | 11.01±0.60 |
|  | S | 41.71±5.38 | 2.39±0.82 | 12.86±16.78 | 39.43±10.80 | 11.05±1.45 |
|  | B | 39.86±13.68 | 2.86±0.90 | 5.29±4.42 | 38.43±6.27 | 11.01±1.60 |
| 18h | C | 65.43±25.80 | 24.00±8.24 | 381.57±134.86 | 39.00±11.27 | 20.92±1.57 |
|  | S | 129.00±53.34 | 19.54±10.23 | 334.86±80.17 | 41.14±3.93 | 28.64±8.3 |
|  | B | 86.00±57.28 | 22.14±7.15 | 414.00±146.10 | 46.86±7.88 | 29.88±11.61 |
| 24h | C | 102.71±60.20 | 34.91±10.06 | 396.00±90.11 | 44.14±14.01 | - |
|  | S | 145.33±77.54 | 33.97±15.93 | 345.00±60.49 | 37.67±15.38 | - |
|  | B | 114.71±79.69 | 30.57±7.14 | 411.43±172.90 | 46.00±7.77 | - |

Abbreviations: ALT, aspartate aminotransferase; TB, total bilirubin; TBA, total bile acids; CR, creatinine; PT, prothrombin time.

**Supplementary Table. S2**

**The efficacy of each PLS-DA model**

| Groups | Component | R2X(cum) | R2Y(cum) | Q2(cum) |
| --- | --- | --- | --- | --- |
| C0 VS. C24 | 1 | 0.527 | 0.986 | 0,972 |
| B24 VS. C24 | 2 | 0.408 | 0.953 | 0.731 |
| B24 VS. S24 | 3 | 0.519 | 0.99 | 0.704 |
